# Proportions of Surgical Patients Discharged Home the Same or the Next Day Are Sufficient Data to Assess Cases’ Contributions to Hospital Occupancy

**DOI:** 10.7759/cureus.13826

**Published:** 2021-03-11

**Authors:** Franklin Dexter, Richard H Epstein, Pengyi Shi

**Affiliations:** 1 Anesthesiology, University of Iowa, Iowa City, USA; 2 Anesthesiology, University of Miami Miller School of Medicine, Miami, USA; 3 Operations Research, Purdue University, West Lafayette, USA

**Keywords:** coronavirus 2019, operating room management, hospital length of stay, clinical classifications software

## Abstract

Introduction

When the hospital census is high, perioperative medical directors or operating room (OR) managers may need to consider postponing some surgical cases scheduled to be performed within the next three workdays. This scenario has arisen at hospitals in regions with large increases in admissions due to coronavirus disease 2019 (COVID-19). We compare summary measures for hospital length of stay (LOS) to guide the OR manager having to decide which cases may need to be postponed to ensure a sufficient reserve of available inpatient beds.

Methods

We studied the 1,201,815 ambulatory and 649,962 inpatient elective cases with a major therapeutic procedure performed during 2018 at all 412 non-federal hospitals in Florida. The data were sorted by the hospital, and then by procedure category. Statistical comparisons of LOS were made pairwise among all procedure categories with at least 100 cases at (the) each hospital, using the chi-square test (LOS ≤ 1 day versus LOS > 1 day), Student’s t-test with unequal variances, and the Wilcoxon-Mann-Whitney test. The comparisons among the three tests then were repeated having sorted the data by procedure category and making statistical comparisons among all hospitals with at least 100 cases for the procedure category.

Results

Whether using a criterion for statistical significance of P < 0.05 or P < 0.01, and whether compared with Student’s t-test with unequal variances or Wilcoxon-Mann-Whitney test, the chi-square test had greater odds (i.e., greater statistical power) to detect differences in LOS (all four with P* *< 0.0001 and all 95% lower confidence limits for odds ratios ≥ 3.00). The findings were consistent when the data, first sorted by procedure category and then by probability distributions of LOS, were compared between hospitals (all P < 0.0001 and the 95% lower confidence limits for odds ratio ≥ 3.72).

Conclusions

For purposes of comparing procedure categories pairwise at the same hospital, there was no loss of information by summarizing the probability distributions using single numbers, the percentages of cases among patients staying longer than overnight. This finding substantially simplifies the mathematics for constructing dashboards or summaries of OR information system data to help the perioperative OR manager or medical director decide which cases may need to be postponed, when the hospital census is high, to provide a sufficient reserve of inpatient hospital beds.

## Introduction

When the hospital census is high, operating room (OR) managers or perioperative medical directors may need to consider postponing some surgical cases scheduled to be performed within the next three workdays. This scenario has arisen at hospitals in regions with large increases in admissions due to coronavirus disease 2019 (COVID-19). We compare summary measures for hospital length of stay (LOS) to guide decision-making related to which cases may need to be postponed to ensure a sufficient reserve of available inpatient beds.

One summary measure is the percentage of patients previously undergoing the same category of the procedure as that scheduled whose hospital LOS was zero or one day [1,2]. This percentage of patients with LOS ≤ 1 day is simple to understand, being a single number. These are the patients undergoing procedures that can be performed safely on an ambulatory basis [3]. Comparisons of percentages with LOS ≤ 1 day between groups can use the well-known Fisher’s exact test or the chi-square test [4]. These percentages can be estimated accurately in practice from the OR schedule, shown using time-series analyses from a large teaching hospital [5].

A second measure is the mean and standard deviation of the LOS for each category of the procedure [1]. Student’s t-test with unequal variances is robust to deviations from a normal or lognormal distribution [1,6]. However, for interpreting LOS, both the mean and the standard deviation need to be considered, the latter of which can be challenging to interpret [1].

A third measure is a probability that a patient undergoing one category of procedure would have a smaller LOS than a patient undergoing another category. This is the area under the receiver operating characteristic curve, calculated by the Wilcoxon-Mann-Whitney test. Two‑group quantile plots or similar graphs provide equivalent information. This approach is distribution-free, but does not summarize each case; rather, it provides for pairwise comparisons among all cases [7,8].

Previously, we considered statistical analyses of LOS as a secondary endpoint of a randomized clinical trial, with efficacy as the primary endpoint between two groups undergoing thoracoscopic lung lobectomy or wedge resection [1]. A simple comparison of the percentage of cases with LOS ≤ 1 day had no lower statistical power than more complicated methods [1].

In the current study, we used 1 year of data from every surgical case at every non-federal hospital in Florida [2]. We compared LOS among procedure categories at each hospital using the three statistical tests, above. We hypothesized that the statistical power would be at least as large from (1) comparing percentages of patients with LOS ≤ 1 day versus (2) Student’s t-test with unequal variances or (3) Wilcoxon-Mann-Whitney tests. If so, hospital descriptive-analytic tools (i.e., dashboards) could rely simply on the summary measure of the percentage of cases with LOS ≤ 1 day. That would be useful because the percentage can be estimated accurately in practice from the daily OR schedule [5].

## Materials and methods

The University of Miami Institutional Review Board determined on July 13, 2020, that this research does not meet the regulatory definition of human subjects research.

Elective cases with a major therapeutic procedure performed during 2018 in Florida

We obtained from Florida Health, publicly available data for inpatient hospitalizations and ambulatory surgical procedures between January 1, 2018, and December 31, 2018 [9], subject to a data use agreement dated May 28, 2019. These data included every surgical case at all non-federal hospitals and ambulatory surgery centers in Florida [10]. We henceforth refer to all such facilities as “hospitals;” segmentation between types of facilities does not affect our results and conclusions (see Statistical Analyses section, below). The selections of procedures for analyses were done as described previously for statewide assessments of (a) surgeon cases per day on dates with at least one case and (b) growth in surgeon cases per week from one year to another [11,12].

For the inpatient data, each discharge had listed procedures classified using the International Classification of Diseases, Tenth Revision, Procedure Coding System (ICD‑10-PCS). We included the case if the primary procedure was a major therapeutic procedure (i.e., procedure class = “4”), the date of admission was on the date of the primary procedure, the admission priority was not listed as urgent or emergent, and there were no emergency room charges for the admission. For each of the studied 649,962 cases, the primary ICD-10-PCS code was mapped to the relevant Clinical Classifications Software (CCS) procedure category [2]. These are broad but meaningful categories. For example, CCS #2, “incision and excision of CNS,” includes supratentorial craniotomy and burr holes with aspiration or evacuation of the hematoma.

For the ambulatory surgery data, we excluded the Current Procedural Terminology (CPT) codes that were not for major therapeutic procedures, based on the CPT’s associated surgery flag field having a value of “narrow.” To determine which of the listed ambulatory procedures was functionally primary, we used the April 2018 Physician Fee schedule from the Centers for Medicare and Medicaid Services to calculate for each CPT code its work relative value units and percentage attributed to intraoperative care [13]. We mapped the CPT code with the largest value of operative work (i.e., work relative value units × percent attributed to the OR) to its relevant CCS procedure category. Although there were 1,202,429 ambulatory cases, 614 included transfer with hospital admission, with an unknown length of stay. We excluded those 0.033% of cases, leaving 1,851,777 LOS for analysis, where 1,851,777 = 649,962 inpatient + 1,202,429 ambulatory - 614 missing values.

Statistical analyses

Our primary analyses were to assess the statistical power among three different statistical tests for making comparisons in LOS among procedure categories clustered by the hospital. Analyses performed from the perspective of individual hospitals were of primary interest because an operating room manager at an individual hospital would compare categories of procedures at their hospital to identify those most increasing their hospital’s census, not data from another hospital. We made pairwise comparisons of procedures, with comparisons of LOS only among patients undergoing surgery at the same hospital. To achieve this, the data were sorted by the hospital, and then by procedure category. Comparisons of LOS were made pairwise among all procedure categories with at least 100 cases at the hospital. There were 4089 such combinations comprising 1,464,656 cases. A minimum of 100 cases per year is small, just two cases per week of these broad procedure categories. We used a minimum of 100 cases because we previously showed in simulation studies with N = 100 per each of two groups that Student’s t-test with unequal variances and Wilcoxon-Mann-Whitney test obtained nominal Type I error rates [1]. That means, for a P < 0.05 criterion, when 100 comparisons are made from two identical distributions of LOS, 5% of the comparisons would be expected to be judged to be different at the P < 0.05 threshold. The same applied to P < 0.01, but for 1% of the comparisons. The version of the Student’s t-test with unequal variances used in the current study was that with the Satterthwaite approximation of the variance.

We used the chi-square test instead of Fisher’s exact test because all groups had N ≥ 100, and 4.8% of groups had N ≥ 1000. Because of the large sample sizes, there were multiple instances of numerical overflow when calculating the factorials used in the numerator and denominator in Fisher’s exact test, especially in the creation of the latter two of our three tables. The chi-square test is less conservative than Fisher’s exact test [4] (i.e., not with drawback for our specific application). For the 42,772 comparisons of procedure categories, nested by the hospital, there were 1,464,656 cases. The median (25th, 75th percentiles) of cases per procedure category was 229 (149,403) over the year. To check the Type I error rate, we selected a hospital at random, selected a procedure category at random, sampled 229 cases with replacement from that hospital-procedure combination for one group, then sampled another 229 cases with replacement from that same hospital-procedure combination to create the second group. We repeated the process 100,000 times.

The chi-square test was compared pairwise versus Student’s t-test with unequal variances and versus the Wilcoxon-Mann-Whitney test. These comparisons represented matched case-control binary studies (e.g., the numbers indicated in the top-left cell for the first group of 2 × 2 comparisons in the tables correspond to the number of cases where the chi-square test had P < 0.05 and Student’s t-test with unequal variances had P < 0.05). The pairwise inferential analyses were performed using the McNemar test. The effect size was estimated using the odds ratio and its 95% confidence interval. Both test and confidence interval were calculated using exact methods (Stata 16.1, StataCorp LLC, College Station, TX).

As explained above, throughout the paper, we refer to “hospital,” but we formally studied hospitals and ambulatory surgery centers. The geographic locations, financial relationships, and functional status are ambiguous for some of the hospitals and surgery centers (e.g., formally independent ambulatory surgery centers geographically close enough to the hospital to be an outpatient department) [14,15]. In addition, there are hospitals that for our study were functionally ambulatory surgery centers. For example, one hospital had 1713 cases involving 64 different procedure categories, but only two procedure categories each with at least 100 discharges, both outpatient procedures, and indeed every patient had a LOS of zero days. Our treatment of all facilities as hospitals did not affect results because the McNemar tests and the odds ratios in the tables depend only on the ratio of the off-diagonal terms (i.e., the upper right cell divided by the lower-left cell). This is because hospitals in which all patients have LOS = 0 days only contribute to the diagonal terms, not the off-diagonal terms. P-values, odds ratios, and confidence intervals are thus unaffected even when such hospitals are removed from the analyses.

Secondary analyses were performed with hospitals clustered by procedure categories (i.e., pairwise comparisons of hospitals, with patients compared having undergone the same procedure). This was done to help understand the results of the primary analyses. To achieve this, the data were sorted by procedure categories, and then by the hospital. There were 203,759 comparisons of hospitals nested by procedure, totaling 1,464,946 cases. The median cases per hospital were 238 (148, 423) over the year. There were 8.0% of groups with N ≥ 1000.

## Results

The chi-square test had statistical power that was at least as large as either the Wilcoxon-Mann-Whitney or Student’s t-test with unequal variances to detect differences in LOS between procedure categories, confirming our hypothesis. Our primary analyses are in Table 1. They show that the chi-square test had reliably greater odds of detecting differences in LOS versus the other two tests. For example, consider the comparison of LOS by the chi-square test versus the Wilcoxon-Mann-Whitney test using the P < 0.05 criterion. There were 7851 comparisons of procedure categories for which the chi-square test detected significant differences in LOS and the Wilcoxon-Mann-Whitney failed to detect a difference. There were fewer comparisons, 2136, for which the Wilcoxon-Mann-Whitney test detected differences in LOS between groups, and the chi-square test failed to do so. Taking the ratio of 7851 to 2136, the estimate of the odds ratio equaled 3.68. The corresponding 95% confidence interval for the ratio was 3.50 to 3.86, with P < 0.0001. This greater statistical power was not obtained at the expense of Type I error rates exceeding the nominal level; see the "Statistical analyses" subsection in the Methods and the next paragraph. Thus, for purposes of comparing procedure categories pairwise at the same hospital, there would be no loss of information by summarizing the probability distribution using a single number, the percentage of cases among patients staying longer than overnight. This substantially simplifies the mathematics for constructing a dashboard to assist the OR manager in decision-making about which cases to postpone.

**Table 1:** Detection of significant differences in length of stay among 42,772 pairwise comparisons of categories of procedures using each of three different statistical methods. ^a^This table summarizes the comparisons made within each hospital among all procedure category pairs each comprising at least 100 cases. Each of the four 2 × 2 square counts sum to 42,772, which equals the number of pairwise comparisons among 101 categories of procedures, each with at least 100 cases and nested within one or more of the 412 hospitals. Odds ratios for paired comparisons are computed by dividing the top-right cell in each contingency table by the lower-left cell. For example, for the 2 × 2 square at the top, there were 34,968 comparisons for which the chi-square test obtained P < 0.05 versus 28,382 for which Student t-test did so. The odds ratio equaled 8842/2256 = 3.92; the 8842 = number of comparisons with chi-square test obtaining P < 0.05 while Student t-test with unequal variances did not obtain P < 0.05 and 2256 = number of comparisons with chi-square test not obtaining P < 0.05 while Student t-test did so. When a test was indeterminate (e.g., all lengths of stay are identical for all patients in both groups), no significant difference was attributed to the test. There is no effect on the odds ratio and our results from the 26,126 comparisons with P < 0.05 for both tests or the 5548 comparisons with both tests failing to detect a difference in LOS between procedures. Equivalently, the sum of the four cells at the top equaled 42,772. Among the 42,772 comparisons, there were 412 facilities, and among those 412 were 238 with all cases having LOS of 0 days. Those 238 facilities provided 2592 of the 5548 comparisons. When those facilities were deleted, the P-values, odds ratios, and confidence intervals for the first pair of rows were identical.

	Statistical Method Applied With Odds Ratios for Pairwise Comparisons of Procedures^a^		
	Student t-test with unequal variances P < 0.05		
	P < 0.05	P ≥ 0.05	
Chi-square test P < 0.05	26,126	8842	McNemar test P < 0.0001
Chi-square test P ≥ 0.05	2256	5548	Odds ratio 3.92 (3.74 to 4.11)
	Student t-test with unequal variances P < 0.01		
	P < 0.01	P ≥ 0.01	
Chi-square test P < 0.01	24,554	8929	McNemar test P < 0.0001
Chi-square test P ≥ 0.01	2530	6759	Odds ratio 3.53 (3.38 to 3.69)
	Wilcoxon-Mann-Whitney test P < 0.05		
	P < 0.05	P ≥ 0.05	
Chi-square test P < 0.05	27,117	7851	McNemar test P < 0.0001
Chi-square test P ≥ 0.05	2136	5668	Odds ratio 3.68 (3.50 to 3.86)
	Wilcoxon-Mann-Whitney test P < 0.01		
	P < 0.01	P ≥ 0.01	
Chi-square test P < 0.01	25,778	7705	McNemar test P < 0.0001
Chi-square test P ≥ 0.01	2471	6818	Odds ratio 3.19 (3.00 to 3.26)

One potential undesirable mechanism for the findings would be if the Type I error rate for the chi-square test were greater than the nominal value (e.g., more than 5.00% rejected at the P < 0.05 criterion). We tested this by selecting 229 cases at random (see Methods) and with replacement from each of two randomly created groups of the same procedure category. There were 3.76% and 0.66% rejected at the P < 0.05 and P < 0.01 criteria, respectively. The corresponding standard errors were 0.06% and 0.02%, respectively. Thus, the chi-square test was, in fact, conservative.

**Figure 1:**
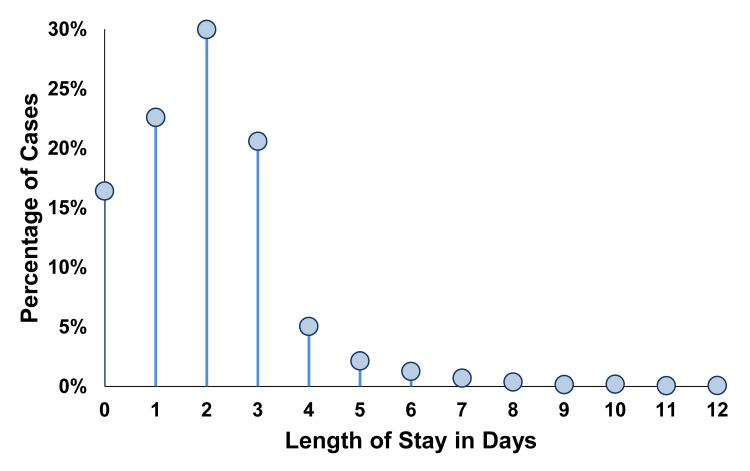
Distribution of lengths of stay. Among the 1,851,777 cases of 138 different procedure categories, there were 65.22% of lengths of stay (LOS) equal to zero days and the mean LOS was 1.55 days. The Clinical Classification Software (CCS) procedure category 152 knee arthroplasty (N = 65,551) has 16.4% LOS equal to zero days and a mean of 2.04 days. Even though both are considerably larger than typical among all cases, the figure shows that the distribution has a little relationship either to normal or log‑normal distribution, the latter considerably so because of the many zero values. The figure does not display the 0.4% with LOS longer than 12 days, but the vertical axis includes all cases. The probability distribution differs markedly from Poisson, based on the sample mean (2.04 days) differing considerably from the sample variance (4.42 days) and the Pearson goodness-of-fit test, P < 0.0001.

We expected that the reason for the findings of Table 1 was that most categories of procedures at the hospitals had few (e.g., ≤ 10%) patients staying two or more nights (Figure 1). To explore this hypothesis, we followed the approach in our previous study [1]. We selected a procedure category and then analyzed all pairwise combinations of hospitals with at least 100 cases per hospital of that procedure. Thus, in our first secondary analysis (Table 2), each of the two groups compared by each test was two hospitals, using the same procedure category. We then repeated for all other procedure categories. For example, a manager may use state data by procedure category to evaluate if their hospital has longer lengths of stay than other hospitals. Longer can be quantified (e.g., in a dashboard) by the percentage of patients staying longer than overnight, by using the nonparametric Wilcoxon-Mann-Whitney approach (e.g., two-group quantile plot), or by using two moments, specifically the estimated mean and standard deviation of LOS. Such knowledge could result in an analysis of factors causing the prolonged LOS and their potential reduction.

**Table 2:** Detection of significant differences in length of stay among 203,759 pairwise comparisons of hospitals using each of three different statistical methods. ^a^This table summarizes the comparisons of LOS made between hospitals using the same procedure categories, with at least 100 cases in each category at the compared hospitals. Each of the four 2 × 2 square counts sum to 203,759, which equals the number of pairwise comparisons among 484 hospitals, each with at least 100 cases and nested within one or more of the 92 categories of procedures. Odds ratios for paired comparisons are computed by dividing the top-right cell in each contingency table by the lower-left cell. For example, for the 2 × 2 square at the top, the ratio equaled 54,023/13,102 = 4.12; the numerator 54,023 = number of comparisons with chi-square test obtaining P < 0.05 while Student t-test with unequal variances did not obtain P < 0.05 and the denominator 13,102 = number of comparisons with chi-square test not obtaining P < 0.05 while Student t-test did so. There were more cases per group for Tables 2 than for Table 1 because there were 448 hospitals included in at least one of the 203,759 comparisons in Tables 2, more than the 101 procedure categories included in at least one of the 42,772 comparisons in Table 1.

	Statistical Method Applied With Odds Ratios for Pairwise Comparisons of Procedures^a^		
	Student t-test with unequal variances P < 0.05		
	P < 0.05	P ≥ 0.05	
Chi-square test P < 0.05	52,104	54,023	McNemar test P < 0.0001
Chi-square test P ≥ 0.05	13,102	84,530	Odds ratio 4.12 (4.04 to 4.20)
	Student t-test with unequal variances P < 0.01		
	P < 0.01	P ≥ 0.01	
Chi-square test P < 0.01	44,530	50,931	McNemar test P < 0.0001
Chi-square test P ≥ 0.01	13,439	94,859	Odds ratio 3.80 (3.72 to 3.86)
	Wilcoxon-Mann-Whitney test P < 0.05		
	P < 0.05	P ≥ 0.05	
Chi-square test P < 0.05	54,322	51,805	McNemar test P < 0.0001
Chi-square test P ≥ 0.05	11,917	85,715	Odds ratio 4.35 (4.26 to 4.43)
	Wilcoxon-Mann-Whitney test P < 0.01		
	P < 0.01	P ≥ 0.01	
Chi-square test P < 0.01	47,502	47,959	McNemar test P < 0.0001
Chi-square test P ≥ 0.01	12,349	95,949	Odds ratio 3.88 (3.81 to 3.96)

Table 2 shows the same result qualitatively as Table 1. Comparing the proportions of stays longer than one day has at least as large statistical power to detect differences among hospitals; in fact, it has greater statistical power. We have rejected the hypothesis that including more information (and complexity) for a dashboard is providing increased useful information, just more numbers or pictures.

The importance of Table 2 is that because hospitals are nested under the procedure category, we could repeat the calculations for interpretable subsets of the population. In our additional secondary analysis (Table 3), we show the results when partitioning the 2 × 2 contingency table comparing the performance of the chi-square test with the Wilcoxon-Mann-Whitney test, based on the P < 0.01 criterion. The partitioning was done based on non-overlapping ranges for each procedure category’s observed percentage of cases with patients having outpatient surgery or staying overnight. The sum of the 20 cells in Table 3 matches that of the corresponding four cells in Table 2. That is the meaning of having partitioned the contingency table.

**Table 3:** Comparing hospitals pairwise using Wilcoxon-Mann-Whitney test and chi-square test based on P < 0.01 criterion, partitioned based on procedure categories’ percentages of cases with hospital lengths of stay zero or one day. ^a^The 1,464,949 cases included in this table are the same as those in Table 2. In addition, the sum of the five 2 × 2 square counts equals 203,759, the number of pairwise comparisons between hospitals in Table 2. (Specifically, 47502 + 47959 + 12349 + 95949 from the last two rows of Table 2 equals, from this table, 75 + 662 + 81 + 41501 + 16502 + 38487 + 2941 + 43596 + 22285 + 6049 + 2912 + 7353 + 6145 + 2152 + 1520 + 1458 + 2495 + 609 + 4895 + 2041.) Odd ratios for paired comparisons are computed by dividing the top right cell in each contingency table by the lower left cell. For example, for the 2 × 2 square at the top, the ratio equaled 662/81 = 8.17; the 662 = number of comparisons with chi-square test obtaining P < 0.01 while Wilcoxon-Mann-Whitney test did not obtain P < 0.01 and 81 = number of comparisons with chi-square test not obtaining P < 0.01 while Wilcoxon-Mann-Whitney did so. There were more cases per group for Tables 3 than for Table 1 because there were 448 hospitals included in at least one of the 203,759 comparisons in Tables 3, more than the 101 procedure categories included in at least one of the 42,772 comparisons in Table 1. ^b^An example of procedure categories with few cases statewide having LOS ≤ 1 day is Cesarean section, Clinical Classifications Software (CCS) #134, specifically 433/45,689. The chi-square test performed worse than Wilcoxon-Mann-Whitney based on P < 0.01, odds ratio 0.07 (0.06 to 0.08). The results for the other tests were 0.14 (0.12 to 0.15) for Student t-test with unequal variances based on P < 0.05, 0.11 (0.09 to 0.12) for t-test based on P < 0.01, and 0.10 (0.08 to 0.11) for Wilcoxon-Mann-Whitney test based on P < 0.05.

	Statistical Method Applied With Odds Ratios for Pairwise Comparisons of Hospitals^a^		
	Procedure categories with > 99% of cases statewide having lengths of stay ≤ 1 day (the 22 procedure categories account for 31% of the cases in Table 2, specifically 457,390)		
	Wilcoxon-Mann-Whitney P < 0.01	Wilcoxon-Mann-Whitney P ≥ 0.01	
Chi-square test P < 0.01	75	662	McNemar test P < 0.0001
Chi-square test P ≥ 0.01	81	41,501	Odds ratio 8.17 (6.48 to 10.4)
	Procedure categories with ≤ 99% but > 80% of cases statewide having lengths of stay ≤ 1 day (the 24 procedure categories account for 29% of the cases in Table 2, specifically 425,747)		
	Wilcoxon-Mann-Whitney P < 0.01	Wilcoxon-Mann-Whitney P ≥ 0.01	
Chi-square test P < 0.01	16,502	38,487	McNemar test P < 0.0001
Chi-square test P ≥ 0.01	2941	43,596	Odds ratio 13.09 (12.6 to 13.6)
	Procedure categories with ≤ 80% but > 35% of cases statewide having lengths of stay ≤ 1 day (the 21 procedure categories account for 20% of the cases in Table 2, specifically 295,075)		
	Wilcoxon-Mann-Whitney P < 0.01	Wilcoxon-Mann-Whitney P ≥ 0.01	
Chi-square test P < 0.01	22,285	6049	McNemar test P < 0.0001
Chi-square test P ≥ 0.01	2912	7353	Odds ratio 2.08 (1.99 to 2.17)
	Procedure categories with ≤ 35% but > 10% of cases statewide having lengths of stay ≤ 1 day (the 12 procedure categories account for 10% of the cases in Table 2, specifically 141,000)		
	Wilcoxon-Mann-Whitney P < 0.01	Wilcoxon-Mann-Whitney P ≥ 0.01	
Chi-square test P < 0.01	6145	2152	McNemar test P < 0.0001
Chi-square test P ≥ 0.01	1520	1458	Odds ratio 1.42 (1.32 to 1.51)
	Procedure categories with ≤ 10% of cases statewide having lengths of stay ≤ 1 day^b^ (The 22 procedure categories account for 10% of the cases in Table 2, specifically 145,737)		
	Wilcoxon-Mann-Whitney P < 0.01	Wilcoxon-Mann-Whitney P ≥ 0.01	
Chi-square test P < 0.01	2495	609	McNemar test P < 0.0001
Chi-square test P ≥ 0.01	4895	2041	Odds ratio 0.12 (0.11 to 0.14)

Conceptually, it should be that using LOS per se would provide more information than the binary classification of the percentage of cases with a LOS of at most one day. Table 3 shows that this was indeed so for those categories of procedures for which at least 90% of patients had a length of stay longer than one night. What explains the results for Tables 1, 2 is that such procedure categories accounted for only 10% of cases.

## Discussion

Previously, we found that for thoracoscopic lung lobectomy and wedge resection, statistical power to compare two groups was at least as large by comparing percentages of patients with LOS ≤ 1 day than versus Student’s t-test with unequal variances or Wilcoxon-Mann-Whitney tests [1]. In the current study, we showed generalizability of this finding to all major therapeutic procedures, pooled by their procedure category, as relevant to decision-making by OR managers using a dashboard or other electronic tool to review the OR schedule one to three days preoperatively (Table 1). The immediate application of our finding is for bed management of the COVID-19 pandemic when the hospital census is high and there are regional mandates to maintain available beds for such patients [2,5]. The OR manager will have many issues to consider, including the patients’ medical conditions, their residential location and travel time to the hospital, surgeon availability, etc. We found that when balancing multiple competing objectives and making decisions for scores or hundreds of cases per week, the manager can consider which cases to postpone based on the simple statistic of whether there is a high probability that the patient will remain in the hospital two days or longer. The mean LOS alone is insufficient for such decision-making (Figure 1) [1]. Our results show that the manager need not try to interpret the mean and standard deviation of the LOS, a challenging task, especially because the probability distributions of LOS are skewed. For example, among Florida cases, “Cesarean section” had a mean hospital LOS of 2.57 days (N=45,689) [2]. The category “other operating room procedures on vessels other than head or neck” had a similar mean LOS of 2.67 days (N=29,090). Yet, the probability distributions were strikingly different (e.g., 0.9% and 67.8% of patients discharged within one day). The groups’ standard deviations of 1.18 and 6.83 days cannot be neglected and treated as the same. The manager does not need complex tools to compare probability distributions pairwise among all cases, for example, by the Wilcoxon-Mann-Whitney test [7]. Our findings, that consideration of the probability of a LOS > 1 day is sufficient, greatly simplifies the manager’s difficult decision to choose which patients’ surgery to postpone. This is because the percentage can, in practice, be estimated accurately in practice from the daily OR schedule [5].

Three recent studies provide insight into the timing of the application of our work. Epstein et al. examined the time of discharge of all types of patients at hospitals in Florida [16]. Only 20% of discharges were before 12 noon [16]. Most hospitals did not even have 50% of discharges before 3 PM [16]. However, Nelson et al. examined 778 patients with overnight stays in the post-anesthesia care unit [17]. The patients with LOS ≤ 1 day were discharged approximately 24% faster than equivalent patients staying on a hospital ward [17]. Assel et al. examined the postoperative length of stay of patients being discharged the day after surgery [18]. Three quarters were discharged between 10 AM and 12 noon [18]. The implication would be that among hospitals with limited census and principally performing surgery among patients being discharged on the day of surgery or the day after surgery, the OR manager can suitably evaluate cases daily in the mid-afternoon for two days hence.

Consider patients scheduled for surgery today. For each, from their CCS procedure category (i.e., scheduled CPT or ICD-10-PCS codes), there is an estimate for the probability of them staying two days or longer. The expected value of the sum of independent Bernoulli trials (i.e., the binary result of LOS ≤ 1 day versus LOS > 1 day) equals the sum of the individual probabilities. (For example, suppose that there were 50 cases, 25 of one category of procedure and 25 with another. The estimated probabilities of the patients staying longer than overnight equal 0.5% and 3.5%, respectively. Then, the estimated mean numbers of patients with LOS > 1 day equals 1.0, where 1.0 = 25 × 0.005 + 25 × 0.035.) Therefore, an estimate for the total number of patients staying two days or longer would be the sum of the individual probabilities [19]. Lower and upper prediction intervals can similarly be calculated just from the estimated probabilities (e.g., as needed for short-term adjustments to nurse staffing, while balancing costs). This is shown in Mønsted et al.’s Appendix S2, wherein the Poisson binomial is well approximated by the Gaussian distribution [19]. From the sum of the individual probabilities, the manager would have a reasonable estimate for the number of beds two days after surgery that would be attributable to the OR schedule [19]. These calculations also can be applied by hospitals contemplating long-term changes to the master surgical schedule to even out admissions and the use of hospital beds among weekdays. Fügener et al. published a tutorial with an example from a surgical suite [20]. Some contemporary studies treat each patient as having a length of stay equal to the mean for their procedure category (i.e., assuming perfect predictive ability) [21-25]. Others use discrete empirical probability distributions, statistically analogous to the Wilcoxon-Mann-Whitney approach [20,26-29]. What our results show is that for postoperative hospital beds pooled for each downstream location, the impact of the OR schedule on bed use can be summarized simply by using the probability of the patient remaining hospitalized for at least two days following surgery.

Strengths of our study are our consideration of every category of major therapeutic procedures and the appropriate limitation to elective surgery. However, our results are limited to the 90% of patients undergoing a major therapeutic procedure that has itself a < 90% probability of remaining in the hospital two days or longer (Table 3). However, we doubt that would be important, practically. At a hospital concerned about high hospital occupancy, no mathematics needs to be applied to know a patient scheduled to undergo complex cardiac surgery will remain hospitalized for more than one day. Our results and conclusions are relevant to the patients for whom it is unclear whether they will remain in the hospital more than one day (Table 3). We conservatively limited our conclusion as showing that when comparing LOS among patients undergoing elective surgery at a hospital, the chi-square test performs at least as well, nominally, as more complex methods using information about the probability distributions.

## Conclusions

For purposes of comparing procedure categories pairwise at the same hospital, there is no loss of information by summarizing the probability distributions using single numbers, the percentages of cases among patients staying longer than overnight. This finding substantially simplifies the mathematics for constructing dashboards or summaries of OR information system data to help the OR manager or perioperative medical director decide which cases may need to be postponed to keep a sufficient reserve of inpatient hospital beds due to a high hospital census.
